# Organizational and social work environment factors, occupational balance and no or negligible stress symptoms among Swedish principals – a cross-sectional study

**DOI:** 10.1186/s12889-021-10809-6

**Published:** 2021-04-26

**Authors:** Carita Håkansson, Ulf Leo, Anna Oudin, Inger Arvidsson, Kerstin Nilsson, Kai Österberg, Roger Persson

**Affiliations:** 1 Division of Occupational and Environmental Medicine, Lund University, Medicon Village, SE-223 81 Lund, Sweden; 2 Centre for Principal Development, Umeå University, Umeå, Sweden; 3 Department of Psychology, Lund University, Lund, Sweden

**Keywords:** Gothenburg Manager Stress Inventory, Lund University Checklist for Incipient Exhaustion, Mental health, Psychosocial work environment, School leaders, Work-life balance

## Abstract

**Background:**

Few studies have assessed the mental health of principals, or studied associations with both organizational and social work environment factors and occupational balance. The purpose of the present study was therefore to investigate associations between supporting and demanding organizational and social work environment factors, occupational balance and stress symptoms in principals.

**Methods:**

A total of 4309 surveys (2316 from the first round, 1992 from the second round), representing 2781 Swedish principals who had responded to at least one of two surveys, were included in the present study. The surveys include questions about socio-demographic factors, occupational balance, overtime work, and supporting and demanding organizational and social work environment factors, as well as questions about personal stress and exhaustion. Generalized Estimating Equations (GEE) models were used to specify a repeated measures model with a dichotomous outcome (binary logistic regression) and multiple independent factors. Data from two surveys were combined, taking into account dependent observations due to the fact that many study subjects had participated in both surveys.

**Results:**

Associations were found between occupational balance (Q1: OR 2.52, 95% CI 2.03–3.15; Q2: OR 4.95, 95% CI 3.86–6.35; Q3: OR 9.29, 95% CI 6.99–12.34), overtime work (Once a week: OR 1.51, 95% CI 1.10–2.08; Sometimes a week: OR 1.31, 95% CI 1.03–1.66), supportive private life (OR 1.50, 95% CI 1.36–1.66), supportive colleagues at the leadership level (OR 1.24, 95% CI 1.14–1.36), supportive management (OR 1.17, 95% CI 1.07–1.28) and no or negligible stress symptoms. In addition, role demands (OR 0.72, 95% CI 0.63–0.83), having a container function (OR 0.72, 95% CI 0.64–0.82), collaboration with employees (OR 0.77, 95% CI 0.66–0.89), role conflicts (OR 0.75, 95% CI 0.66–0.89) and having a buffer function (OR 0.86, 95% CI 0.77–0.97) were associated with lower likelihood to rate no or negligible stress symptoms.

**Conclusions:**

The occupational balance of principals is strongly associated with no or negligible stress symptoms, and thus is a promising venue for promoting well-being. Improvements should be made to several factors in the organizational and social work environments to improve principals’ chances of having occupational balance, and therefore better mental health.

## Introduction

International research has highlighted the importance of principals for the development of schools and for student outcomes [1, 2] and it has been shown that leadership is crucial for both teachers’ [3] and students’ health [4]. This focus on educational leadership has put new and demanding expectations on principals, which in turn put principals at risk of health problems [5]. To be able to improve student outcomes and the well-being of students’, teachers’ and principals’ it is necessary to understand the factors promoting the well-being of principals.

Principals are both educational leaders and managers of interpersonal relationships and resources, and they must handle internal as well as external demands. Different stakeholders (parents, teachers, and students) with potentially unreasonable expectations must be managed, which makes this job highly demanding. Furthermore, they have to manage a large volume of paper work, insufficient time to complete jobs, constant interruptions, and a struggle to keep up with communications [6]. Role conflict and work overload were associated with mental health problems (i.e. burnout) in principals [7], who can also be challenged by a diverse student population that often needs social problems to be addressed [8]. The association between on the one hand organizational and social work environmental factors and on the other hand job strain and mental health problems is well established [9], and it has also been shown that principals who do not feel supported by colleagues at leadership levels feel more stress [10]. Previous studies of other populations have shown that occupational balance is important for health and well-being [11, 12] and since principals seem to experience poor work-life balance [13], it is important to study their occupational balance in addition to organizational and social work environment in order to identify factors that make them feel good. Occupational balance is defined as an individual’s subjective experience of having the right amount of work, the right amount of home and family activities, the right amount of leisure for rest, recovery and sleep, and the individual’s subjective satisfaction with the variation among these activities [14]. In the present study, the concept of occupational balance was preferred over the concept of ‘work-life’ balance that focuses on work and how life outside work (especially family life) can be balanced to cope with work [15].

Occupational balance is studied in general populations [16], different patient groups [17, 18], different professions [19, 20] but not among managers. Therefore we merged principals and assistant principals in the present study.

What we have been able to find, there are no studies of the associations between organizational and social work environment, occupational balance and no or negligible stress symptoms among principals.

Therefore, the aim of the present study was to investigate these associations.

## Method

The present cross-sectional study is part of a longitudinal study on the work environment and health of principals, conducted by researchers at Lund University and Umeå University [21]. Data was collected twice with web surveys 1 year apart.

### Participants

There is no accessible official register of principals in Sweden. Therefore, a purposeful sampling was used of principals who had participated in training programs funded and arranged by the Swedish National Agency for Education between 2008 and 2017. Using this list, 9900 presumptive principals were invited in 2018 by email to participate in the present study. Of these, 4640 respondents either accepted (*n* = 2633) or declined (*n* = 1987) participation and 2317 completed the first round questionnaire. The response rate, based on the principals reached was 50%. Precisely 1 year after that first survey, 2316 respondents were asked to complete the second round (one of the former participants had retired). In total, 1528 (66%) completed the second survey, 202 individuals could not be reached (their email bounced, we got automatic replies that they had quit the job, etc.), 42 do not want to participate, and 544 did not respond at all. Those who did not answer the first survey in 2018 got the second survey, and of those 464 principals answered, for a total 1992 respondents to the second round survey in 2019.

The present study includes both principals and assistant principals working at least 50% of full-time in pre-schools, compulsory schools, upper secondary schools, or adult education. In the results, the categories principal and assistant principal were merged. A total of 4309 surveys (2316 from the first round, 1992 from the second round), representing 2781 Swedish principals who had responded to at least one of two surveys, were included in the present study.

### Data collection

Data were collected with the software Texttalk Websurvey (www.texttalk.se, Gothenburg, Sweden) between 25 September and 23 October 2018 and between 24 September and 22 October 2019. A reminder to fill in the survey was sent on four occasions both times. Questions addressed signs of exhaustion, supporting and demanding organizational and social work environment factors, occupational balance, and various socio-demographic factors.

### Outcome

#### Stress symptoms and exhaustion

Stress symptoms and exhaustion were measured with the Lund University Checklist for Incipient Exhaustion (LUCIE) [22]. LUCIE has been shown to have positive associations with other measures in which exhaustion is a core component such as the Karolinska Exhaustion Disorder Scale (KEDS), Self-reported Exhaustion Disorder (S-ED) and the Shirom Melamed Burnout Questionnaire [22].

LUCIE consists of 28 items in 6 domains:

1. Sleep and recovery,
2. Separation between work and leisure,
3. Sense of community and social support at the workplace,
4. Managing work duties and personal ability,
5. Private life and leisure activities and
6. Health complaints.

Each domain consists of questions in the form “For the past month, to what extent have you felt or observed the following?” A four-point response scale is used, ranging from not at all (1) to very much (4). The LUCIE includes the Stress Warning Scale (SWS) and the Exhaustion Warning Scale (EWS). The scores on each scale range from 0 to 100. For a detailed description of the separate algorithms that generate the scores on the SWS and EWS scales, see Persson et al. [22].

##### SWS reflects milder signs of exhaustion

A SWS of score ≤ 17.00 (the SWS green zone) indicate no or negligible lasting stress symptoms, while a SWS score between 17.01 and 38.50 (the SWS yellow zone) suggests possible mild lasting stress symptoms, and a SWS score ≥ 38.51(the SWS red zone) indicates moderate lasting stress symptoms.

##### EWS reflects more severe signs of exhaustion

A EWS score of ≤21.50 (the EWS green zone) indicates that signs of exhaustion are absent or negligible, while a higher EWS score of > 21.50 (the EWS red zone) suggests severe signs of exhaustion that might indicate exhaustion disorder.

These two scales are combined into four steps indicating the severity of the stress:

- Step 1-SWS green zone and EWS green zone = no or negligible lasting stress symptoms
- Step 2-SWS yellow zone and EWS green zone = possible mild lasting stress symptoms
- Step 3-SWS red zone and EWS green zone = moderate lasting stress symptoms, but less severe than exhaustion disorder, and
- Step 4-SWS red zone and EWS red zone = lasting stress symptoms of a severity indicating possible exhaustion disorder [23].

Preliminary analyses of the LUCIE scores verified previous cross-sectional observations [21] showing that around half the principals had no or negligible lasting stress symptoms, approximately 25% had possible mild lasting stress symptoms, approximately 15% had moderate lasting stress symptoms, and approximately 10% had lasting stress symptoms of a severity indicating possible exhaustion disorder. Accordingly, the LUCIE scores were dichotomized into 1 = no or negligible stress symptoms (i.e., LUCIE step 1), and 0 = mild, moderate, or severe stress symptoms indicating exhaustion disorder (i.e., LUCIE steps 2–4).

### Independent factors

#### Supporting and demanding organizational and social work environment factors

Supporting and demanding organizational and social work environment factors were measured with a version of the Gothenburg Manager Stress Inventory (GMSI, [24]), shortened by one of the authors (KÖ). The selected items are those that in two different samples showed the highest correlation with the respective subscale in the original GMSI. This version is referred to as GMSI-mini for the rest of the paper. GMSI-mini measures supportive factors (supportive management, collaboration with employees, supportive colleagues, supportive privacy, supportive organizational resources) and the response alternatives are Applies very poorly (1), Applies poorly (2), Applies to some extent (3), Applies well (4), and Applies very well (5). Higher mean scores for supporting factors indicate that the respondent perceives more support.

GMSI-mini also measures demanding factors (resource imbalance, organizational deficiencies, logic conflicts, burdensome role requirements, group dynamic problems, employee problems, having a buffer function and a container function) with the response alternatives Never/almost never (1), Rarely (2), Sometimes (3), Often (4) and Always/almost always (5).

The mean score of each scale was used as an outcome. Higher mean scores for demanding factors indicate that demands occur more frequently.

The GMSI-mini was tested in a pilot study comprising 253 principals, and it was found that all factors, except organizational deficiencies, had good internal consistency (Cronbach’s alpha > 0.7) [25].

### Occupational balance

Occupational balance was measured with the Occupational Balance Questionnaire (OBQ11) [26]. The OBQ11 consists of 11 statements (Table 1). The OBQ11 has four response categories, ranging from not agreeing at all (0) to fully agreeing (3). The total sum score (0–33) shows the level of occupational balance, from low to high, with higher scores indicating a better level of occupational balance. The OBQ11 has shown to have good reliability (Pearson separation index 0.92), and sufficient construct validity, and with no items differing according to age or gender [26]. For purposes of analysis, the total sum score was divided into quartiles.

**Table 1:** The items in the occupational balance questionnaire (*n* = 4309)

Items (short form)	Median	Quartiles Q1, Q3
1. Having sufficient to do during a regular week	1	0, 1
2. Balance between doing things for myself and for others	1	0, 1
3. Time for doing the things I want	1	1, 1
4. Balance between work, home, family, leisure, rest and sleep	1	1, 1
5. Enough time for obligatory occupations	1	0, 1
6. Balance between physical, social, mental and restful occupations	1	1, 1
7. Satisfaction with how time is spent in everyday life	1	0, 1
8. Satisfaction with the number of occupations during a regular week	1	1, 1
9. Balance between obligatory and voluntary occupations	1	1, 1
10. Balance between energy-giving and energy-taking occupations	1	1, 1
11. Satisfaction with the time spent in rest, recovery and sleep	1	0, 1

The items are responded to on a four step scale (0–3): Strongly disagree [0]; disagree [1]; agree [2]; strongly agree [3]

### Socio-demographic factors

Socio-demographic factors related to occupational balance and health such as years of experience as principal, overtime work, gender, presence of children under 8 years old (yes vs. no), living with another adult person (yes vs. no) and physical activity were analysed. Overtime work was divided into Every day vs. Once a week vs. Some times a week vs. Seldom (sometimes a year/once a month/sometimes a month) vs. Non regulated working hours. Physical activity was measured with the question “How physically active have you been the last three months?”, dichotomised into mainly sedentary versus physically active (light, moderate or heavy) [27].

### Statistical analysis

Frequencies and percentages were used to describe sociodemographic factors whereas quartiles were used to describe the distribution of occupational balance (OBQ11). To investigate the associations between supporting and demanding organizational and social work environment factors, occupational balance, and no or negligible stress symptoms, Generalized Estimating Equations (GEE) models were used to specify a repeated measures model with a dichotomous outcome (binary logistic regression) and multiple independent factors. Data from the initial 2018 survey were combined with data from the second survey in 2019, taking into account dependent observations due to the fact that many study subjects had participated in both surveys. Data are presented as odds ratios (ORs) with their 95% confidence intervals (CIs), meaning that *p*-values < 0.05 were considered as statistically significant. First, univariate analysis was done and independent factors with *p*-values < 0.1 were selected for a multivariate analysis [28] and entered at the same time. Because of some internal missing values, the number of subjects may slightly differ for statistical models. Potential multicollinearity was assessed by investigating bivariate correlations between independent factors and the correlation coefficients were not too great (< 0.7). All statistical analyses were performed using SPSS version 26.0 software.

## Results

The great majority of the principals in the present study were women, most of which had worked as a principal 5–10 years. More than half of them had worked overtime sometimes a week during the last 12 months. Most of the principals lived with someone, but without children under 8 years old. The great majority were physical active (Table 1).

The participants’ occupational balance (OBQ11) scores varied in both the first and second surveys, from 0 to 33 with a median of 11, indicating that the principals on average tended to disagree, or slightly disagree, with the statements in the OBQ11 (Table 1): that is, they were experiencing low occupational balance. In both surveys, half of the principals had no or negligible lasting stress symptoms (LUCIE), which can be interpreted as they felt well (Table 2).

**Table 2:** Characteristics of the participants in 2018 and 2019

	2018 (*n* = 2317) n (%)	2019 (*n* = 1992) n (%)
*Gender*^a^		
Women	1803 (78)	1564 (79)
Men	510 (22)	425 (21)
*Living with someone*		
Yes	1969 (85)	1691 (85)
No	260 (11)	221 (11)
Living apart	88 (4)	80 (4)
*Children under 8 years old*		
Yes	301 (13)	222 (11)
No	2016 (87)	1770 (89)
*Years of experience as a principal*		
< 1 year – 3 years	445 (20)	160 (8)
> 3 years – 5 years	519 (22)	461 (23)
> 5 years – 10 years	797 (34)	791 (40)
> 10 years – 20 years	457 (20)	480 (24)
> 20 years	99 (4)	100 (5)
*Overtime*		
Non regulated working hours	107 (5)	93 (5)
Seldom	273 (12)	300 (15)
Once a week	220 (9)	237 (12)
Some times a week	1284 (55)	1049 (52)
Everyday	433 (19)	313 (16)
*Physical active*		
No	263 (11)	190 (10)
Yes	2054 (89)	1802 (90)
*Occupational balance*		
Q0 (0–7)	681 (29)	514 (26)
Q1 (8–11)	638 (28)	553 (28)
Q2 (12–16)	464 (20)	385 (19)
Q3 (17–33)	534 (23)	540 (27)

^a^Four persons in 2018 and three in 2019 did not want to specify their gender

Univariate associations were found between all variables and no or negligible lasting stress symptoms except for the variable having children under 8 years old or not (Table 3).

**Table 3:** Univariate associations between work environment factors, occupational balance and no or negligible stress symptoms

	OR	95% CI
*Demanding organisational and social work environment factors*		
Resource imbalance	0.63	0.61–0.67
Organisational governance shortcoming	0.57	0.55–0.61
Role conflicts	0.35	0.33–0.41
Role demands	0.32	0.30–0.37
Group dynamic problems	0.49	0.45–0.55
Buffer function	0.53	0.49–0.57
Employee problems	0.56	0.50–0.61
Container function	0.41	0.41–0.45
*Supporting organisational and social work environment factors*		
Supportive management	1.61	1.49–1.65
Collaboration with employees	1.63	1.49–1.82
Supportive colleagues at leadership level	1.52	1.49–1.65
Supportive private life	2.42	2.23–2.46
Supportive organizational structures	1.78	1.65–2.01
*Occupational balance*		
Quartile 0	ref	
Quartile 1	4.19	3.32–4.95
Quartile 2	10.55	8.17–13.46
Quartile 3	30.20	24.53–36.60
*Years of experience as principal* (few - many)	1.14	1.07–1.22
*Gender* (Woman)	1.28	1.11–1.49
*Overtime*		
Everyday	ref	
Non regulated working hours	2.19	1.54–4.65
Seldom	1.61	3.86–47.34
Once a week	1.14	3.11–22.45
Sometimes a week	2.23	1.84–6.28
*Living with someone*		
Yes	ref	
No	0.81	0.67–1.00
Living apart	1.12	0.82–1.65
*Children under 8 years*		
Yes	ref	
No	1.15	0.50–1.35
*Physical active*		
No	ref	
Yes	2.34	1.86–2.92

### Associations between supporting and demanding organisational and social work environment factors, occupational balance, and having no or negligible stress symptoms

The strongest association was found between *occupational balance* (Q1: OR 2.52, 95% CI 2.03–3.15; Q2: OR 4.95, 95% CI 3.86–6.35; Q3: OR 9.29, 95% CI 6.99–12.34) and no or negligible stress symptoms. Another strong association was found between *working overtime* (once a week (OR 1.51, 95% CI 1.10–2.08) or some times a week (OR 1.31, 95% CI 1.03–1.66) and no or negligible stress symptoms. Furthermore, associations were found between *supportive management* i.e., trust that the manager, if necessary, helps to solve work environment problems and shows interest in what the principal does and what problems there are (OR 1.17, 95% CI 1.07–1.28), *supportive colleagues at leadership level* i.e., opportunities to receive beneficial support from colleagues at leadership level and the opportunity to discuss business with these colleagues (OR 1.24, 95% CI 1.14–1.36) and *a supportive private life* i.e., opportunities to refrain from thinking about work during leisure time and leisure activities that provide rest and relaxation (OR 1.50, 95% CI 1.36–1.66) and no or negligible stress symptoms.

However, in the multivariate analysis, the direction of the odds ratios suggested that some organisational and social work environment factors were associated with less likelihood to rate no or negligible stress symptoms. These factors were *role conflicts* i.e., conflicts between educational development, administration, and contact with employees (OR 0.75, 95% CI 0.66–0.89), *role demands* i.e., responsibility for results, quality, staff, work environment, and development (OR 0.72, 95% CI 0.63–0.83), *having a buffer function* i.e., buffer between higher levels of the organization and the employees, the requirements to explain superiors’ negative decisions to employees, and expectations of superiors that principals should be understanding and accept decisions (OR 0.86, 95% CI 0.77–0.97), *having a container function* i.e., receiving the employees’ frustrations with work that is psychologically stressful, and that pressured employees’ problems are burdensome (OR 0.72, 95% CI 0.64–0.82) and *collaboration with employees* i.e., the employees take responsibility and they have valuable knowledge that facilitates the principal’s work (OR 0.77, 95% CI 0.66–0.89) (Table 4).

**Table 4:** Multivariate associations between work environment factors, occupational balance and no or negligible stress symptoms

	OR	95% CI
*Demanding organisational and social work environment factors*		
Resource imbalance	0.97	0.88–1.06
Organisational governance shortcoming	0.97	0.86–1.09
**Role conflicts**	0.75	0.66–0.86
**Role demands**	0.72	0.63–0.83
Group dynamic problems	0.91	0.79–1.05
**Buffer function**	0.86	0.77–0.97
Employee problems	0.92	0.80–1.05
**Container function**	0.72	0.64–0.82
*Supporting organisational and social work environment factors*		
**Supportive management**	1.17	1.07–1.28
**Collaboration with employees**	0.77	0.66–0.89
**Supportive colleagues at leadership level**	1.24	1.14–1.36
**Supportive private life**	1.50	1.36–1.66
Supportive organizational structures	1.07	0.97–1.18
*Occupational balance*		
Quartile 0	ref	
**Quartile 1**	2.52	2.03–3.15
**Quartile 2**	4.95	3.86–6.35
**Quartile 3**	9.29	6.99–12.34
*Years of experience as principal*		
≤ 3 years	ref.	
> 3 years – 5 years	1.110.97	0.78–1.31
> 5 years – 10 years	1.09	0.76–1.25
> 10 years		0.83–1.43
*Gender*		
Man	ref.	
Woman	0.98	0.80–1.20
*Overtime*		
Everyday	ref	
**Sometimes a week**	1.31	1.03–1.66
**Once a week**	1.51	1.10–2.08
Seldom	1.31	0.95–1.80
Non regulated working hours	0.62	0.38–1.00
*Living with someone*		
Yes	ref	
No	0.86	0.65–1.12
Living apart	1.06	0.70–1.59
*Physical active*		
No	ref	
Yes	1.27	0.96–1.69

## Discussion

About half of the principals in the present study reported no or negligible lasting stress symptoms. Associations were found between occupational balance, overtime work, a supportive private life, supportive colleagues at the leadership level, supportive management and no or negligible stress symptoms. In addition, role demands, having a container function, collaboration with employees, role conflicts and having a buffer function were associated with less likelihood to rate no or negligible stress symptoms.

Taken together, role demands, having a container function, collaboration with employees, role conflicts and having a buffer function can lead to a high work load, which is a known predictor of stress and exhaustion [7, 19]. The principals in the present study struggle to handle demands for educational development, administration, and contact with employees and others, which may cause too high of a work load that may surpass their ability to cope with all these demands, as has been shown previously [29]. Interestingly, the principals in the present study seemed to cope with this high work load by working overtime. In fact, more than half of the principals worked overtime some times a week. Nevertheless, working overtime once or some times a week was associated with an increased likelihood of reporting no or negligible stress symptoms. This result is consistent with previous research [30] that has also shown that working overtime was related to lower stress.

This group of principals seemed to prioritise work over other activities, and it seems reasonable to assume that it will affect their occupational balance by reducing the time and energy the principals have for home and family activities as well as rest, recovery and sleep. The OBQ11 is a rather new measure and does not have a specific cut-off score, making it difficult to assess whether a group’s occupational balance is high or low. However, in view of the scale range (0–33), and the principals’ median score of 11 a reasonable interpretation is that the principals in the present study were experiencing low occupational balance. Furthermore, the quartiles (Q1 and Q3) of the different items in the OBQ11 (Table 1) were rather low, especially concerning item 1, item 2, item 5 and item 11. These items showed that the principals had too much to do, had no balance between doing things for others and for themselves, not had enough time to do obligatory occupations and were not satisfied with the time spent in rest, recovery and sleep. Taken together, they did seem to have neither the right variation between different activities nor the right amount of different activities both of which are necessary for occupational balance [14].

The results further showed that occupational balance had the strongest association with having no or negligible stress symptoms among the principals in the present study. This result confirms previous studies that have shown associations between occupational balance and mental health [12, 16, 18]. Given these results, it seems important that managers support principals in setting limits for their work through for example continuous communication and feedback, prioritising and accessibility guidelines and thus help principals experience occupational balance and hopefully also better mental health. The support of managers has been shown to be crucial for enable occupational balance of principals [31]. Because occupational balance had the strongest association with no or negligible stress symptoms in the present study and, because having occupational balance is better for health than having stress and exhaustion, the occupational balance questionnaire could potentially be used for assessing the progress of various health promotion efforts.

In addition to the factors named above, support from private life, colleagues at leadership level and management were also associated with no or negligible stress symptoms among the principals in the present study. These results partially confirm the results of another study of principals [8], where it was shown that support from colleagues at leadership level predicted decreased stress. That study did not investigate the other types of support but it seems reasonable that both a supportive private life and supportive management are also important for preventing stress symptoms in principals. Furthermore, without a supportive private life, it would probably not have been possible to use overtime work to alleviate stress.

Another interesting result, though difficult to interpret, was that the GMSI-factor “collaboration with employees” was associated with lower likelihood of having no or negligible stress symptoms. In other word, increased collaboration seems to be associated with increased stress. In the present study, to collaborate with employees mean that employees were taking responsibility and they had valuable knowledge that facilitated the principals’ work. A possible interpretation could be that employees who collaborated in this way resulted in the principal feeling more connected with them, and perhaps this connection took time and energy, which in the end, increased work load and was perceived as stressful. Another interpretation may be that when employees took a lot of responsibility, demands on principal increased, which then increased principal’s stress. However, these speculations will have to be further studied qualitatively.

Supporting and demanding organisational and social work environment factors and occupational balance were factors associated with no or negligible stress symptoms among principals in the present study. These results partially corroborated those of De Young et al. [13], who showed that high job demands, unreasonable demands from different stakeholders, lack of support, and lack of work-life balance were sources of job dissatisfaction.

## Strenghts and limitations

A strength in the present study is that principals from all 21 Swedish Regions participated in the study, and the mean age, gender distribution, and years of experience of these principals was representative of principals in general in Sweden [32], which strengthened the external validity. The large study sample provided good statistical power, which strengthened the internal validity of the study. The data were gathered at exactly the same time period on both occasions, which decreased the risk that yearly fluctuations in work load differently affected the two measurement points, which strengthened internal validity.

The study has limitations as well. Even if the data were collected longitudinally, the analyses are cross-sectional, which limited causal interpretations between study variables. Furthermore, some participants took part once, and some twice, which was taken account for in the analysis, by using a repeated measurements statistical model. However, if there were trends in answers, depending on if it was the first (or only) or second time a person participated, this was not taken into account for in the analysis. In that mode, the two surveys are considered independent in the analysis, which might be a limitation. Another limitation is the rather low response rate, only 26% (*n* = 2317) of all 9900 invited individuals completed the first survey. However, of the 4640 principals we reached about 50% selected to complete the survey. The absence of an official register made us use the selection strategy recruiting from an email list that had accumulated over a 10 year period, and within that time some principals may have left their employment. The second survey was answered by 1992 principals, and some of whom had not answered first survey. These response rates are in line with other large-scale studies in Sweden. This sample is also representative of Swedish principals concerning age and gender [33]. Another limitation is that the psychometric properties of the shortened form of the GMSI has not been evaluated, which may decrease the internal validity of the present study. Another possible limitation is that we merged principals and assistant principals and did not adjust for stage but our main interest was to study managers. We are aware of that these two groups may have different roles and in future studies, it is preferable to study them separately and adjust for different stages.

## Conclusion

High occupational balance is strongly associated with no or negligible stress symptoms among principals, and thus is a promising area to focus on for promoting their well-being. Improvements should be made to several factors in the organizational and social work environments to improve principals’ chances of having occupational balance, and therefore better mental health.

## Data Availability

The dataset used and analysed during the current study is available from the corresponding author on reasonable request.

## Abbreviations

GEE: Generalized Estimating Equations
GMSI: Gothenburg Manager Stress Inventory
EWS: Exhaustion Warning Scale
LUCIE: Lund University Checklist for Incipient Exhaustion
OBQ11: Occupational Balance Questionnaire 11
SWS: Stress Warning Scale
